# A new method to disperse CdS quantum dot-sensitized TiO_2_ nanotube arrays into P3HT:PCBM layer for the improvement of efficiency of inverted polymer solar cells

**DOI:** 10.1186/1556-276X-9-240

**Published:** 2014-05-16

**Authors:** Fumin Li, Chong Chen, Furui Tan, Gentian Yue, Liang Shen, Weifeng Zhang

**Affiliations:** 1 Key Laboratory of Photovoltaic Materials, Department of Physics and Electronics, Henan University, Kaifeng 475004, People's Republic of China; 2 Department of Electronic Science and Engineering, Jilin University, Changchun 130012, People's Republic of China

**Keywords:** Inverted, Polymer solar cells, Nanotube, Quantum dot

## Abstract

We report that the efficiency of ITO/nc-TiO_2_/P3HT:PCBM/MoO_3_/Ag inverted polymer solar cells (PSCs) can be improved by dispersing CdS quantum dot (QD)-sensitized TiO_2_ nanotube arrays (TNTs) in poly (3-hexylthiophene) and [6,6]-phenyl-C_61_-butyric acid methyl ester (P3HT:PCBM) layer. The CdS QDs are deposited on the TNTs by a chemical bath deposition method. The experimental results show that the CdS QD-sensitized TNTs (CdS/TNTs) do not only increase the light absorption of the P3HT:PCBM layer but also reduce the charge recombination in the P3HT:PCBM layer. The dependence of device performances on cycles of CdS deposition on the TNTs was investigated. A high power conversion efficiency (PCE) of 3.52% was achieved for the inverted PSCs with 20 cyclic depositions of CdS on TNTs, which showed a 34% increase compared to the ITO/nc-TiO_2_/P3HT:PCBM/MoO_3_/Ag device without the CdS/TNTs. The improved efficiency is attributed to the improved light absorbance and the reduced charge recombination in the active layer.

## Background

Polymer solar cells (PSCs) have gained great interest because of their low cost, flexibility, and abundant availability [1-7]. So far, the high power conversion efficiency (PCE) of PSCs is achieved by bulk heterojunction (BHJ) PSCs composed of electron-donating polymers and electron-accepting fullerides [8]. Although significant progress has been made on the improvement of the PCE of PSCs in recent years, the efficiency of the PSCs is still lower than their inorganic counterparts, such as silicon and CIGS. The main factors limiting the efficiency of the PSCs are the low light absorption efficiency due to the narrow absorption band of the absorption spectra of the polymers and the charge recombination in the devices due to the low charge transport efficiency in the electron-donating and electron-accepting materials [9]. To overcome these problems, many efforts have been made on improving the absorption spectra and charge carrier mobility of the photovoltaic materials for higher PCE [10-13]. Some inorganic nanostructure materials with high light absorption of the visible spectrum and the near infrared spectral range are dispersed in to the polymer:fulleride layer to increase the light absorption such as CdS [14,15], CdSe [16], PbS [17], Sb_2_S_3_[18], and FeS_2_[19,20]. In addition, some inorganic materials with high charge carrier mobility, such as ZnO and TiO_2_, are used to increase the charge transport efficiency and reduce the charge recombination [21-23]. Specially, because the ordered TiO_2_ nanotube arrays (TNTs) possess outstanding charge transport properties, the TNTs are used to reduce the charge recombination in the PSCs and therefore improved the efficiency as reported recently [24]. It is worthy to note that most of these materials are synthesized in advance through complicated chemical method and then dispersed in active layers. Of which, usually, only one type of these inorganic nanostructure materials is dispersed in active layer. However, there are few reports on which two types of inorganic nanostructure materials are compactly combined and dispersed in active layers.

This report focuses on the synthesis of the CdS quantum dot (QD)-sensitized TiO_2_ nanotube arrays (CdS/TNTs) in a simple way (chemical bath deposition (CBD)) and dispersion in active layers. CdS QDs help light absorption to produce more excitons and also help to form the interface of CdS/P3HT with P3HT in the P3HT:PCBM layer so that more excitons are separated. TNTs are able to make prompt transfer of the excitons produced by light absorption of CdS QDs. Excitons are separated efficiently enough to reduce the charge recombination. Meanwhile, TNTs are used to form the interface of TNTs/P3HT with P3HT in the active layer and also enhance the separation of excitons. Therefore, CdS/TNTs synthesized using the CBD method and dispersed in P3HT:PCBM layer not only increase the light absorption but also reduce the charge recombination. It is known that few studies on the synthesis of CdS/TNTs using the CBD method to enhance PSCs' PCE are reported.

The result shows that after the CdS/TNTs are dispersed in the P3HT:PCBM layer, the light absorption of the active layer is greatly improved, and the charge recombination is largely controlled. Comparing to the device without CdS/TNTs, the efficiency of the device with CdS/TNTs mentioned above increases by 34%, which fully proves the reasonability of this reported method.

## Methods

### Fabrication of TNTs

Highly ordered and vertically oriented TNTs were prepared by anodization of Ti (titanium foil, 0.25-mm thickness, 99.7% purity; Sigma-Aldrich, St. Louis, MO, USA) sheets in an electrolyte consisting of 0.25 wt.% ammonium fluoride (NH_4_F) (98 + % purity; Sigma-Aldrich) and 0.5 wt.% distilled (DI) water in ethylene glycol (EG) (C_2_H_6_O_2_, 99.0% purity; Sigma-Aldrich) at 40 V for 8 h. A detailed experimental procedure has been described in our previous paper [25]. After anodization, the samples were washed with DI water to remove the occluded ions and dried in a N_2_ stream. Finally, the samples were annealed at 450°C for 2 h with a heating rate of 5°C min^-1^ at ambient conditions.

### Synthesis of CdS-coated TNTs

CdS as an inorganic photon absorption material was deposited on TNTs by sequential CBD. Briefly, the as-prepared TNTs were successively immersed in four different beakers for about 40 s each: beakers contained a 50 mM cadmium chloride (CdCl_2_) (98.0%; Sigma-Aldrich) aqueous solution and a 50 mM sodium sulfide nonahydrate (Na_2_S) (98.0% purity; Sigma-Aldrich) aqueous solution, respectively, and the other two contained DI water to wash the samples to remove the excess of each precursor. The CBD process was performed by dipping the prepared TNTs in CdCl_2_ aqueous solution, rinsing it with DI water, dipping it in Na_2_S aqueous solution, followed by a further rinsing with DI water. The two-step dipping procedure is considered as one CBD cycle. After several cycles, the sample became yellow. In this study, 10, 20, and 30 cycles of CdS deposition were performed (denoted as CdS(10), CdS(20), and CdS(30), respectively). The as-prepared samples were dried in a N_2_ stream. The TNT sample after *n* cycles of CdS deposition was denoted as CdS(*n*)/TNTs. Finally, the CdS(*n*)/TNT powder was peeled off from the Ti sheets by bending them.

### Fabrication of devices

**Figure 1:**
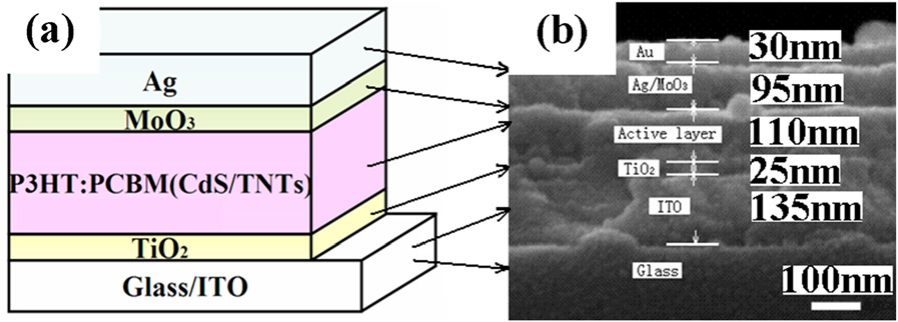
**Structure and SEM cross-sectional image of the inverted polymer solar cell. (a)** Schematic structure drawing of the inverted polymer solar cell. **(b)** The SEM cross-sectional image of the device corresponding with the drawing of the structure. Scale bar = 100 nm.

The photovoltaic device has a structure of ITO/nc-TiO_2_/P3HT:PCBM (CdS/TNTs)/MoO_3_/Ag (P3HT, 95 + % regioregular, electronic grade, Luminescence Technology Co., Hsin-Chu, Taiwan; PCBM, 99.5 + %, Luminescence Technology Co.) as shown schematically in Figure 1a. The ITO-conducting glass substrate (a sheet resistance of 15 Ω/□) was pre-cleaned using acetone, ethanol, and DI water for 15 min each. Anatase phase TiO_2_ thin films was prepared as described in our previous papers [26,27]. The thickness of TiO_2_ is 25 nm. P3HT (used as received) was dissolved in 1,2-dichlorobenzene to produce an 18-mg/ml solution, followed by blending with PCBM (used as received) in 1:1 weight ratio [28]. The blend was divided into four equal parts after being stirred for 72 h in air. Then, the same quality of CdS(*n*)/TNTs (*n* = 10, 20, 30) powder was dispersed in the blend to produce a 1-mg/ml solution, respectively. Simultaneously, there was one equal part which did not contain CdS(*n*)/TNTs (denoted as CdS(0)/TNTs). The blend was ultrasonically disrupted for 2 h in air and then was continuously stirred before spin coating on top of the TiO_2_ film surface. Then, the samples were baked in low vacuum (vacuum oven) at 150°C for 10 min. The typical film thickness of P3HT:PCBM (CdS(*n*)/TNTs) was about 100 nm. Finally, 1 nm of MoO_3_ and 100 nm of Ag were thermally evaporated in sequence under high vacuum (5 × 10^-4^ Pa) without disrupting the vacuum. The deposition rate was about 0.05 nm/s, which was monitored with a quartz-oscillating thickness monitor (CRTM-9000, ULVAC, Methuen, MA, USA). These thicknesses could be observed from Figure 1b that shows the cross-section of a typical device by scanning electronic microscope (SEM). It was noticed that there was about 30 nm of Au sputtered on the surface of the sample, as seen on SEM. The active area of the device was about 4 mm^2^.

### Characterization and measurements

Current density-voltage (*J*-*V*) characteristics were measured using a computer-programmed Keithley 2400 sourcemeter (Cleveland, OH, USA) under AM1.5G solar illumination using a Newport 94043A solar simulator (Jiangsu, China). The intensity of the solar simulator was 100 mW/cm^2^. Light intensity was corrected by a standard silicon solar cell. The transmission and reflection spectra were measured using ultraviolet/visible (UV-vis) spectrometer (Cary 5000, Agilent Technologies Inc., Santa Clara, CA, USA).

## Results and discussion

**Figure 2:**
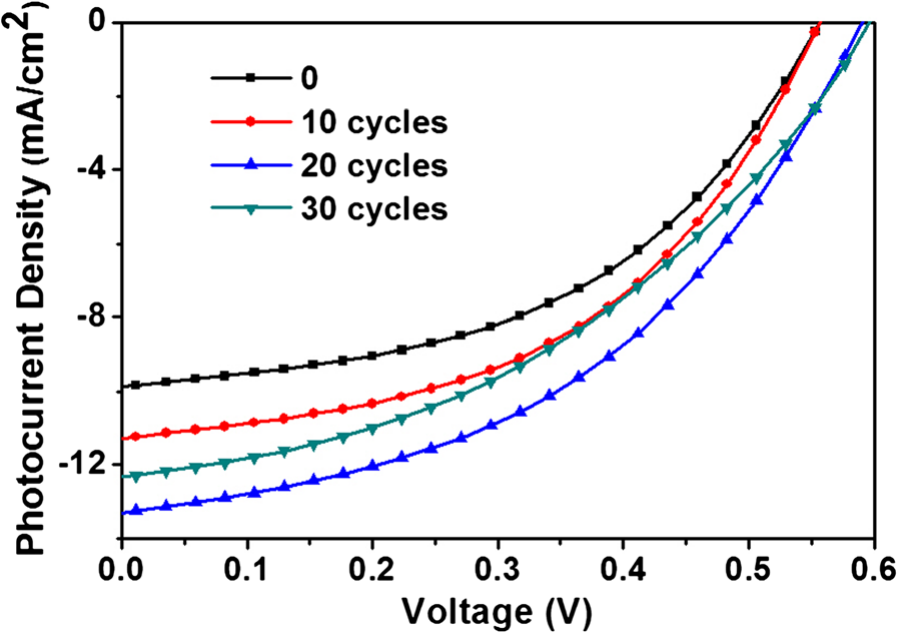
***J*-*V *characteristics of the device.** The characteristics depend on the number of cycles of CdS deposition which is varied from 0 to 30 times under AM1.5G illumination of 100 mW/cm^2^.

**Figure 3:**
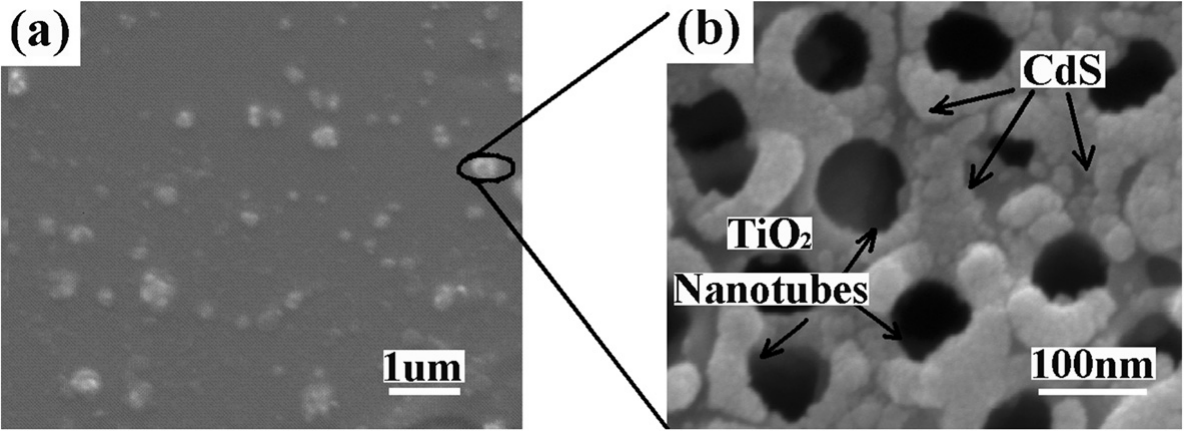
**SEM surface image of a typical device. (a)** The SEM surface image of a typical device; scale bar, 1 μm. **(b)** Regional enlargement image of the CdS/TNTs; scale bar, 100 nm.

Figure 2 shows the *J*-*V* characteristics of the inverted PSCs when cycles of CdS deposition vary from 0 to 30 times under AM1.5G illumination of 100 mW/cm^2^. The detailed results are given in Table 1. The control sample device (without CdS(*n*)/TNTs) shows a short-circuit current density (*J*sc) of 9.84 mA/cm^2^, open-circuit voltage (*V*oc) of 0.56 V, fill factor (FF) of 48.12%, and PCE of 2.63%. When the CdS depositions are 20 cycles, the photovoltaic device has a *J*sc of 13.31 mA/cm^2^, *V*oc of 0.56 V, FF of 48.81%, and PCE of 3.52%. The *J*sc of the device with 0 cycles is the smallest, and the *J*sc of the device with 20 cycles is the largest. It shows a 34% efficiency increase compared to the control sample device. It is possible for the limited absorbing ability of P3HT:PCBM. When depositing CdS(*n*)/TNT powder in the blend, the performance has improved remarkably because of its good light absorption properties and electron transport capacity. When the CdS deposition is 30 cycles, the *J*sc of the photovoltaic device reduces to 12.28 mA/cm^2^, while FF and PCE reduce as well. It can be interpreted that the bigger size of the CdS/TNT powders rather than the fewer cycles can depress their degree of dispersion in the blend after too many depositions. As a result, the film formation of the device is not good, and the series resistance of the device increases. It is well known that the series resistance greatly affects the fill factor and efficiency of solar cells [16]. The main characteristic parameters are slightly reduced.To investigate whether the CdS/TNTs are evenly dispersed in the blend, the surface SEM images of a typical device is shown in Figure 3 at different scale bars. Figure 3a shows the image of the device at a scale bar of 1 μm. It can be clearly seen that after ultrasonic disruption and magnetic stirring, PCBM blended with the CdS/TNT powders, and then after spin coating, the CdS/TNT powders are distributed on the surface of active layer. To see the details, Figure 3b shows the regional enlargement image of the CdS/TNTs at a scale bar of 100 nm. The CdS is well coated on the surface of the TNTs. The two types of inorganic nanostructure materials are compactly combined and dispersed in active layers uniformly.

**Table 1:** Characteristic data of inverted polymer solar cells with different cycles of CdS deposition on TNTs

**Cycles**	***J***_**SC **_**(mA/cm**^**2**^**)**	***V***_**OC **_**(V)**	**FF (%)**	**PCE (%)**	***R*** **s (Ω)**
0	9.84	0.56	48.12	2.63	32.6
10	11.29	0.56	47.63	3.01	33.5
20	13.31	0.59	48.81	3.52	30.2
30	12.28	0.60	41.13	3.04	44.9

**Figure 4:**
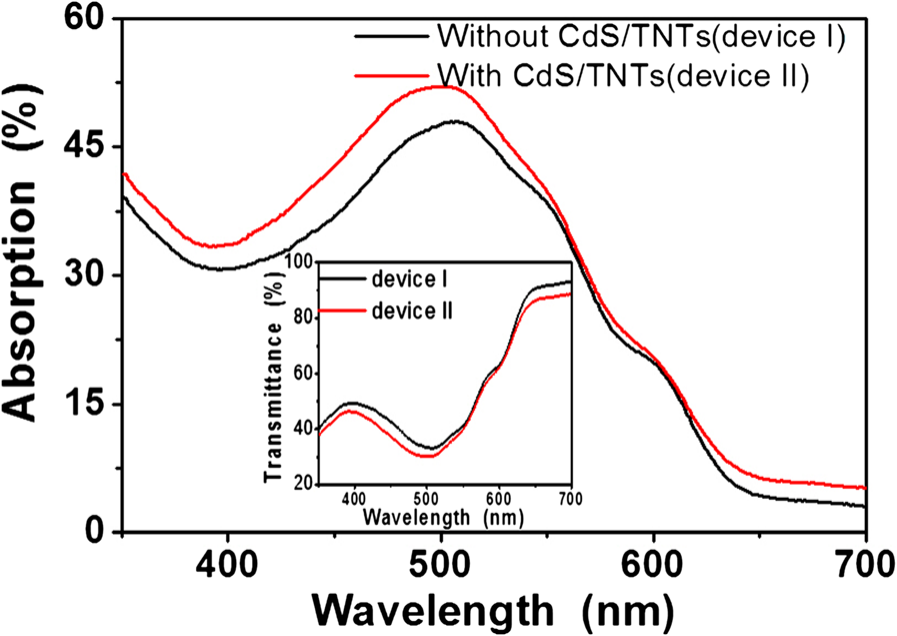
**Absorption for the two devices with and without the CdS(*n*)/TNTs.** The inset is the corresponding transmission spectra of the two devices between the wavelength 350 and 700 nm.

Figure 4 shows the UV-vis absorption spectra and the corresponding transmission spectra of the inverted PSCs with 20 cycles (device II) and without CdS(*n*)/TNTs (device I) between the wavelengths 350 and 700 nm. Obviously, after the CdS(*n*)/TNTs deposition, the absorption of the device II films appears around 400 to 650 nm. The absorbance of the spectra of the CdS(*n*)/TNTs films increases significantly not only in the UV region but also in the visible region, which is mainly due to the CdS(*n*)/TNT light absorption within the 350- to 500-nm excitation spectral range. It can be seen that the device II has a wider absorption range and a stronger absorption intensity than device I. CdS/TNTs are suitable for absorption enhancement of photovoltaic application.

**Figure 5:**
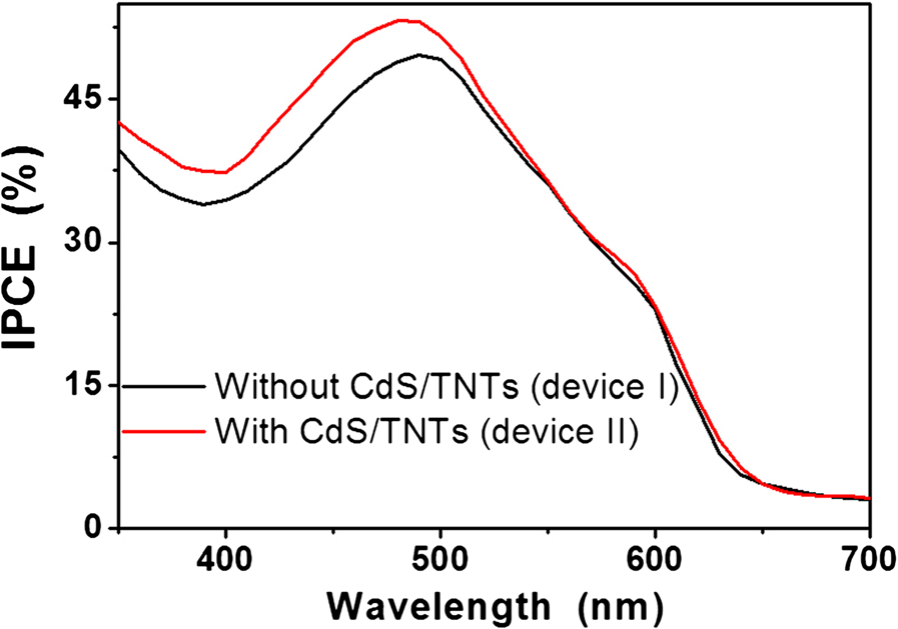
**IPCE for the two devices with and without the CdS(*n*)/TNTs.**

Figure 5 compares the incident photon-to-current collection efficiency (IPCE) spectrum of devices fabricated with and without the CdS(*n*)/TNT deposition in the active layer. The IPCE is defined as the number of photo-generated charge carrier contributing to the photocurrent per incident photon. The conventional device (without the CdS(*n*)/TNTs) shows the typical spectral response of the P3HT:PCBM composites with a maximum IPCE of approximately 50% at 500 nm, consistent with the previous studies [29,30]. For device II (with the CdS(*n*)/TNTs), the results demonstrate a substantial enhancement of approximately 10% in the IPCE less than the 500 nm excitation spectral range. The reason for this phenomenon may be due to the increased light absorption, which can be seen from Figure 4. On one hand, the increased light absorption due to the introduction of the CdS/TNT powder led to more generated electrons. On the other hand, the introduction of the CdS/TNT powders resulted in a larger exciton-dissociation interface area, such as CdS/P3HT and TNTs/P3HT interfaces, which may lead to a more efficient dissociation efficiency of excitons generated in the active layer.

## Conclusions

In summary, we demonstrated a new method which significantly improves the solar cells' efficiency which could be obtained via simply dispersing compactly combined CdS/TNTs in an active layer. The CdS/TNTs were synthesized by sequential chemical bath deposition. As a result, a high PCE of 3.52% was achieved for the inverted PSCs with 20 cycles of CdS, which showed a 34% increase compared to conventional P3HT:PCBM devices. We believe that this is a simple but effective method that can be used to improve the efficiency of polymer solar cells.

## Competing interests

The authors declare that they have no competing interests.

## Authors' contributions

FL carried out the experiments, participated in the sequence alignment, and drafted the manuscript. CC participated in the device preparation. FT, GY, LS, and WZ were involved in the SEM, UV-vis, and IPCE analysis of the devices. All authors read and approved the final manuscript.
